# Supplementary material for: Task-evoked pulse wave amplitude tracks cognitive load
Source: Sci Rep. 2023 Dec 18;13:22592. doi: 10.1038/s41598-023-48917-5 (PMC10730617; doi:10.1038/s41598-023-48917-5)
Supplement: Supplementary file 1 — Supplementary Information. [file 41598_2023_48917_MOESM1_ESM.pdf]

Supplementary materials

Table S1 – Pairwise comparison of relative pulse wave amplitude in 2 second time windows after digit presentation averaged over all sequences. Note – uncorrected p-values are reported.

Contrast (digit serial positions)	Estimate	SE	*t (77)*	*p*
		**Control task**		
1 - 2	-0.01414	0.003424	-4.12953	9.14E-05
1 - 3	-0.03716	0.005871	-6.32941	1.50E-08
1 - 4	-0.0587	0.0082	-7.1582	4.16E-10
1 - 5	-0.07396	0.009783	-7.56018	7.12E-11
1 - 6	-0.08309	0.010704	-7.76312	2.90E-11
1 - 7	-0.09147	0.011376	-8.04085	8.49E-12
1 - 8	-0.09359	0.011334	-8.25756	3.24E-12
1 - 9	-0.0902	0.011654	-7.74006	3.22E-11
1 - 10	-0.07859	0.012787	-6.14637	3.26E-08
1 - 11	-0.07936	0.012164	-6.52438	6.51E-09
1 - 12	-0.07301	0.012294	-5.939	7.80E-08
1 - 13	-0.06963	0.012559	-5.54452	3.98E-07
2 - 3	-0.02302	0.003215	-7.16065	4.12E-10
2 - 4	-0.04456	0.005983	-7.44706	1.17E-10
2 - 5	-0.05982	0.007858	-7.61277	5.64E-11
2 - 6	-0.06895	0.009179	-7.5122	8.79E-11
2 - 7	-0.07733	0.010046	-7.69718	3.89E-11
2 - 8	-0.07945	0.010337	-7.68585	4.09E-11
2 - 9	-0.07606	0.010643	-7.14673	4.38E-10
2 - 10	-0.06445	0.011765	-5.47817	5.21E-07
2 - 11	-0.06522	0.01138	-5.73091	1.85E-07
2 - 12	-0.05887	0.01154	-5.10134	2.36E-06
2 - 13	-0.05549	0.011809	-4.69895	1.12E-05
3 - 4	-0.02154	0.003263	-6.60121	4.68E-09
3 - 5	-0.0368	0.005511	-6.67874	3.35E-09
3 - 6	-0.04593	0.007394	-6.21255	2.46E-08
3 - 7	-0.05431	0.008299	-6.54443	5.98E-09
3 - 8	-0.05643	0.00872	-6.47142	8.18E-09
3 - 9	-0.05304	0.009077	-5.84407	1.16E-07
3 - 10	-0.04143	0.010506	-3.94393	0.000175
3 - 11	-0.0422	0.01038	-4.06556	0.000115
3 - 12	-0.03585	0.010473	-3.42313	0.000995
3 - 13	-0.03247	0.010831	-2.99815	0.003656

4 - 5	-0.01526	0.002854	-5.34891	8.80E-07
4 - 6	-0.0244	0.005419	-4.50159	2.36E-05
4 - 7	-0.03277	0.006294	-5.20658	1.55E-06
4 - 8	-0.03489	0.006888	-5.06516	2.72E-06
4 - 9	-0.03151	0.007304	-4.31374	4.71E-05
4 - 10	-0.01989	0.009414	-2.1132	0.037824
4 - 11	-0.02066	0.009563	-2.16056	0.03384
4 - 12	-0.01431	0.009666	-1.48072	0.142763
4 - 13	-0.01093	0.010148	-1.07734	0.284694
5 - 6	-0.00913	0.00426	-2.14337	0.035241
5 - 7	-0.01751	0.005088	-3.44059	0.000941
5 - 8	-0.01963	0.005868	-3.34424	0.001277
5 - 9	-0.01624	0.00631	-2.57367	0.011983
5 - 10	-0.00463	0.008731	-0.53025	0.597466
5 - 11	-0.0054	0.009005	-0.59933	0.550712
5 - 12	0.000952	0.009134	0.104278	0.91722
5 - 13	0.004331	0.009559	0.453078	0.651767
6 - 7	-0.00837	0.002368	-3.53685	0.00069
6 - 8	-0.01049	0.00391	-2.68402	0.008902
6 - 9	-0.00711	0.004994	-1.42373	0.158563
6 - 10	0.004502	0.00828	0.543701	0.588218
6 - 11	0.003734	0.008678	0.430329	0.668159
6 - 12	0.010084	0.008749	1.152554	0.252659
6 - 13	0.013462	0.009121	1.475979	0.144027
7 - 8	-0.00212	0.002314	-0.91564	0.362717
7 - 9	0.001265	0.003688	0.34302	0.732518
7 - 10	0.012877	0.007707	1.670751	0.098831
7 - 11	0.012109	0.008192	1.478085	0.143464
7 - 12	0.018458	0.008447	2.185167	0.031918
7 - 13	0.021837	0.008959	2.437331	0.017103
8 - 9	0.003384	0.002423	1.396921	0.16645
8 - 10	0.014996	0.007279	2.060088	0.042769
8 - 11	0.014228	0.00777	1.831201	0.070939
8 - 12	0.020578	0.008041	2.558982	0.012459
8 - 13	0.023956	0.008549	2.80207	0.00642
9 - 10	0.011612	0.006691	1.735379	0.086674
9 - 11	0.010844	0.007229	1.500026	0.137697
9 - 12	0.017193	0.007703	2.232064	0.028518
9 - 13	0.020572	0.008455	2.433242	0.017283
10 - 11	-0.00077	0.00284	-0.27032	0.787638
10 - 12	0.005582	0.004599	1.213821	0.228526
10 - 13	0.00896	0.006416	1.396573	0.166555
11 - 12	0.006349	0.003448	1.841399	0.069414
11 - 13	0.009728	0.005652	1.721252	0.089221
12 - 13	0.003379	0.00326	1.036427	0.303247

Memory task				
1 - 2	0.003119	0.003019	1.03318	0.304754
1 - 3	0.000858	0.004873	0.176022	0.860739
1 - 4	-0.00219	0.006323	-0.34584	0.730405
1 - 5	0.003589	0.007595	0.472478	0.637922
1 - 6	0.017439	0.009476	1.840337	0.069572
1 - 7	0.042186	0.010607	3.977105	0.000156
1 - 8	0.063826	0.011851	5.385565	7.59E-07
1 - 9	0.081418	0.012635	6.443963	9.20E-09
1 - 10	0.091695	0.013305	6.891786	1.33E-09
1 - 11	0.091649	0.013735	6.672689	3.44E-09
1 - 12	0.084093	0.013811	6.088995	4.15E-08
1 - 13	0.074938	0.014273	5.250226	1.31E-06
2 - 3	-0.00226	0.002403	-0.94083	0.349732
2 - 4	-0.00531	0.004189	-1.26645	0.20917
2 - 5	0.00047	0.005848	0.08033	0.936183
2 - 6	0.01432	0.008156	1.755721	0.083113
2 - 7	0.039067	0.009686	4.033295	0.000128
2 - 8	0.060708	0.011228	5.406758	6.96E-07
2 - 9	0.078299	0.012225	6.404589	1.09E-08
2 - 10	0.088576	0.013069	6.777343	2.19E-09
2 - 11	0.08853	0.013607	6.506407	7.04E-09
2 - 12	0.080974	0.013752	5.888018	9.65E-08
2 - 13	0.071819	0.014079	5.101211	2.36E-06
3 - 4	-0.00304	0.002155	-1.41291	0.161712
3 - 5	0.002731	0.004097	0.666509	0.507078
3 - 6	0.016581	0.00682	2.431144	0.017376
3 - 7	0.041328	0.008685	4.758696	8.94E-06
3 - 8	0.062969	0.010466	6.016559	5.63E-08
3 - 9	0.08056	0.011684	6.894965	1.31E-09
3 - 10	0.090838	0.012663	7.173437	3.90E-10
3 - 11	0.090792	0.013326	6.813338	1.87E-09
3 - 12	0.083236	0.013482	6.173906	2.90E-08
3 - 13	0.07408	0.013726	5.396986	7.25E-07
4 - 5	0.005775	0.002413	2.393918	0.019103
4 - 6	0.019626	0.005426	3.61702	0.00053
4 - 7	0.044372	0.007503	5.913949	8.66E-08
4 - 8	0.066013	0.009531	6.925854	1.15E-09
4 - 9	0.083605	0.010985	7.610785	5.69E-11
4 - 10	0.093882	0.012122	7.744896	3.15E-11
4 - 11	0.093836	0.012915	7.265635	2.60E-10
4 - 12	0.08628	0.013091	6.590855	4.90E-09
4 - 13	0.077124	0.013315	5.79228	1.44E-07
5 - 6	0.01385	0.003522	3.932172	0.000183
5 - 7	0.038597	0.005764	6.695707	3.11E-09

5 - 8	0.060238	0.008005	7.52494	8.31E-11
5 - 9	0.077829	0.009741	7.989646	1.06E-11
5 - 10	0.088107	0.011045	7.976923	1.13E-11
5 - 11	0.088061	0.011974	7.354611	1.76E-10
5 - 12	0.080505	0.012241	6.576458	5.21E-09
5 - 13	0.071349	0.01247	5.721777	1.92E-07
6 - 7	0.024747	0.002931	8.443765	1.42E-12
6 - 8	0.046388	0.005732	8.093321	6.72E-12
6 - 9	0.063979	0.007876	8.122845	5.90E-12
6 - 10	0.074256	0.009559	7.768439	2.84E-11
6 - 11	0.07421	0.010729	6.916639	1.20E-09
6 - 12	0.066654	0.011149	5.978322	6.61E-08
6 - 13	0.057499	0.01145	5.021656	3.23E-06
7 - 8	0.021641	0.003314	6.529452	6.37E-09
7 - 9	0.039233	0.005759	6.812553	1.88E-09
7 - 10	0.04951	0.007855	6.302665	1.68E-08
7 - 11	0.049464	0.009178	5.389291	7.47E-07
7 - 12	0.041908	0.009754	4.296396	5.02E-05
7 - 13	0.032752	0.010251	3.195111	0.002027
8 - 9	0.017592	0.002896	6.07542	4.40E-08
8 - 10	0.027869	0.005664	4.92043	4.79E-06
8 - 11	0.027823	0.007056	3.943336	0.000176
8 - 12	0.020267	0.007831	2.587947	0.011536
8 - 13	0.011111	0.008604	1.29135	0.200446
9 - 10	0.010277	0.004049	2.538227	0.01316
9 - 11	0.010231	0.005312	1.926177	0.057773
9 - 12	0.002675	0.006289	0.425373	0.671752
9 - 13	-0.00648	0.007249	-0.89399	0.374111
10 - 11	-4.61E-05	0.002315	-0.01989	0.984181
10 - 12	-0.0076	0.003886	-1.95619	0.054069
10 - 13	-0.01676	0.005321	-3.14942	0.002329
11 - 12	-0.00756	0.002341	-3.22803	0.001832
11 - 13	-0.01671	0.004065	-4.11067	9.78E-05
12 - 13	-0.00916	0.00251	-3.64833	0.000478

Table S2 – Pairwise comparison of relative heart rate in 2 second time windows after digit presentation averaged over all sequences. Note – uncorrected p-values are reported.

Contrast (digit serial positions)	Estimate	SE	*t (77)*	*p*
		**Control task**		
1 - 2	0.539574	0.103127	5.232144	1.40E-06
1 - 3	1.115654	0.137747	8.099311	6.55E-12
1 - 4	1.462867	0.154542	9.465799	1.52E-14
1 - 5	1.830278	0.14695	12.45507	3.90E-20
1 - 6	2.159818	0.166749	12.95248	5.01E-21
1 - 7	2.427863	0.182738	13.28607	1.29E-21
1 - 8	2.497191	0.171465	14.56381	7.98E-24
1 - 9	2.630301	0.178426	14.74173	4.00E-24
1 - 10	2.345458	0.213265	10.99788	1.88E-17
1 - 11	2.56682	0.230934	11.11496	1.13E-17
1 - 12	2.75577	0.227824	12.09606	1.75E-19
1 - 13	2.687528	0.227044	11.83703	5.21E-19
2 - 3	0.57608	0.094485	6.097074	4.02E-08
2 - 4	0.923293	0.125251	7.371539	1.63E-10
2 - 5	1.290703	0.130291	9.906337	2.18E-15
2 - 6	1.620244	0.150572	10.76062	5.23E-17
2 - 7	1.888289	0.174106	10.84562	3.62E-17
2 - 8	1.957617	0.157567	12.42399	4.44E-20
2 - 9	2.090727	0.169117	12.3626	5.74E-20
2 - 10	1.805883	0.195767	9.224671	4.43E-14
2 - 11	2.027245	0.223527	9.069337	8.82E-14
2 - 12	2.216196	0.218513	10.14218	7.75E-16
2 - 13	2.147954	0.221425	9.7006	5.40E-15
3 - 4	0.347213	0.07888	4.401794	3.41E-05
3 - 5	0.714623	0.105644	6.764438	2.31E-09
3 - 6	1.044164	0.136087	7.672772	4.33E-11
3 - 7	1.312209	0.14368	9.132871	6.65E-14
3 - 8	1.381537	0.130088	10.62002	9.63E-17
3 - 9	1.514647	0.14315	10.58083	1.14E-16
3 - 10	1.229803	0.185752	6.620672	4.31E-09
3 - 11	1.451165	0.201284	7.209543	3.33E-10
3 - 12	1.640116	0.198026	8.282321	2.91E-12
3 - 13	1.571874	0.20844	7.541139	7.74E-11
4 - 5	0.367411	0.071011	5.173983	1.77E-06
4 - 6	0.696952	0.110282	6.319725	1.56E-08
4 - 7	0.964997	0.12452	7.749728	3.08E-11
4 - 8	1.034324	0.118935	8.696513	4.61E-13

4 - 9	1.167434	0.124745	9.358583	2.45E-14
4 - 10	0.882591	0.175868	5.018497	3.27E-06
4 - 11	1.103953	0.19152	5.764178	1.61E-07
4 - 12	1.292904	0.193908	6.667619	3.52E-09
4 - 13	1.224662	0.194484	6.296964	1.72E-08
5 - 6	0.329541	0.101191	3.256622	0.001678
5 - 7	0.597586	0.116003	5.151453	1.94E-06
5 - 8	0.666913	0.124826	5.342758	9.02E-07
5 - 9	0.800024	0.115834	6.906615	1.25E-09
5 - 10	0.51518	0.173479	2.969697	0.003973
5 - 11	0.736542	0.188183	3.913963	0.000195
5 - 12	0.925493	0.181822	5.0901	2.47E-06
5 - 13	0.857251	0.185134	4.63043	1.46E-05
6 - 7	0.268045	0.080029	3.349367	0.001256
6 - 8	0.337372	0.101671	3.318268	0.001385
6 - 9	0.470483	0.099305	4.737746	9.68E-06
6 - 10	0.185639	0.160745	1.154869	0.251716
6 - 11	0.407001	0.17486	2.327577	0.022563
6 - 12	0.595952	0.181921	3.275886	0.001581
6 - 13	0.52771	0.189252	2.788394	0.006671
7 - 8	0.069327	0.092595	0.748717	0.456309
7 - 9	0.202438	0.092871	2.179766	0.032331
7 - 10	-0.08241	0.175393	-0.46984	0.639801
7 - 11	0.138956	0.169984	0.817467	0.416184
7 - 12	0.327907	0.174897	1.874857	0.064604
7 - 13	0.259665	0.184969	1.403831	0.164389
8 - 9	0.13311	0.08082	1.646991	0.103635
8 - 10	-0.15173	0.16623	-0.91279	0.364202
8 - 11	0.069629	0.174792	0.398351	0.691474
8 - 12	0.25858	0.171395	1.508673	0.135475
8 - 13	0.190338	0.176439	1.078775	0.284056
9 - 10	-0.28484	0.157107	-1.81305	0.073721
9 - 11	-0.06348	0.171804	-0.3695	0.712768
9 - 12	0.125469	0.169282	0.741183	0.460837
9 - 13	0.057227	0.173383	0.330062	0.742249
10 - 11	0.221362	0.124016	1.784949	0.078208
10 - 12	0.410313	0.147589	2.780096	0.006827
10 - 13	0.342071	0.145359	2.353279	0.021162
11 - 12	0.188951	0.108531	1.740984	0.085681
11 - 13	0.120709	0.133864	0.901725	0.370015
12 - 13	-0.06824	0.107263	-0.63621	0.526524
**Memory task**				
1 - 2	0.44421	0.111909	3.969375	0.000161
1 - 3	0.65613	0.159732	4.107684	9.88E-05
1 - 4	0.495847	0.207687	2.387479	0.019417

1 - 5	0.354768	0.210255	1.687326	0.095588
1 - 6	0.343891	0.238281	1.443216	0.153016
1 - 7	0.350501	0.254932	1.374883	0.173155
1 - 8	0.187088	0.274613	0.681276	0.497741
1 - 9	0.154395	0.293222	0.526545	0.600023
1 - 10	0.124237	0.305564	0.406583	0.685442
1 - 11	0.284701	0.307888	0.924688	0.358017
1 - 12	0.396767	0.327967	1.209776	0.230066
1 - 13	0.408692	0.335531	1.218046	0.226926
2 - 3	0.21192	0.085754	2.471263	0.015673
2 - 4	0.051637	0.15469	0.333812	0.739428
2 - 5	-0.08944	0.170374	-0.52497	0.60111
2 - 6	-0.10032	0.199254	-0.50347	0.616069
2 - 7	-0.09371	0.210958	-0.44421	0.658139
2 - 8	-0.25712	0.232752	-1.1047	0.272728
2 - 9	-0.28982	0.256664	-1.12917	0.262334
2 - 10	-0.31997	0.280433	-1.141	0.257407
2 - 11	-0.15951	0.283046	-0.56355	0.5747
2 - 12	-0.04744	0.294339	-0.16119	0.872369
2 - 13	-0.03552	0.305126	-0.1164	0.907635
3 - 4	-0.16028	0.093116	-1.72132	0.089208
3 - 5	-0.30136	0.118293	-2.54758	0.01284
3 - 6	-0.31224	0.155492	-2.00807	0.048143
3 - 7	-0.30563	0.166478	-1.83585	0.07024
3 - 8	-0.46904	0.190313	-2.46459	0.015945
3 - 9	-0.50174	0.214777	-2.33608	0.022091
3 - 10	-0.53189	0.244851	-2.17231	0.032909
3 - 11	-0.37143	0.244347	-1.52009	0.132585
3 - 12	-0.25936	0.258018	-1.00522	0.317941
3 - 13	-0.24744	0.272466	-0.90814	0.366636
4 - 5	-0.14108	0.079847	-1.76686	0.081214
4 - 6	-0.15196	0.128159	-1.18569	0.239391
4 - 7	-0.14535	0.147851	-0.98306	0.328659
4 - 8	-0.30876	0.164382	-1.8783	0.064125
4 - 9	-0.34145	0.192811	-1.77092	0.080531
4 - 10	-0.37161	0.227817	-1.63118	0.106936
4 - 11	-0.21115	0.232867	-0.90673	0.367379
4 - 12	-0.09908	0.252989	-0.39164	0.696407
4 - 13	-0.08716	0.268207	-0.32496	0.746096
5 - 6	-0.01088	0.089372	-0.12171	0.903448
5 - 7	-0.00427	0.114235	-0.03735	0.970299
5 - 8	-0.16768	0.134587	-1.2459	0.21658
5 - 9	-0.20037	0.160654	-1.24724	0.216091
5 - 10	-0.23053	0.198968	-1.15864	0.250185
5 - 11	-0.07007	0.201314	-0.34805	0.72875

5 - 12	0.041998	0.227425	0.184669	0.853974
5 - 13	0.053924	0.241223	0.223542	0.823706
6 - 7	0.00661	0.076462	0.086449	0.931334
6 - 8	-0.1568	0.105268	-1.48957	0.140423
6 - 9	-0.1895	0.132622	-1.42884	0.157093
6 - 10	-0.21965	0.173321	-1.26733	0.208858
6 - 11	-0.05919	0.170698	-0.34676	0.729721
6 - 12	0.052876	0.196529	0.269048	0.788612
6 - 13	0.064801	0.216769	0.298939	0.765792
7 - 8	-0.16341	0.060813	-2.68716	0.008826
7 - 9	-0.19611	0.093596	-2.09525	0.039437
7 - 10	-0.22626	0.148128	-1.52749	0.130737
7 - 11	-0.0658	0.146877	-0.448	0.655412
7 - 12	0.046266	0.170961	0.270621	0.787406
7 - 13	0.058191	0.186085	0.312711	0.755346
8 - 9	-0.03269	0.064746	-0.50494	0.615041
8 - 10	-0.06285	0.133528	-0.47069	0.639193
8 - 11	0.097613	0.143321	0.681081	0.497864
8 - 12	0.209679	0.163433	1.282968	0.203352
8 - 13	0.221604	0.173469	1.277488	0.205268
9 - 10	-0.03016	0.105372	-0.2862	0.775494
9 - 11	0.130306	0.121865	1.069269	0.288289
9 - 12	0.242372	0.147915	1.638587	0.105379
9 - 13	0.254297	0.149064	1.705966	0.092045
10 - 11	0.160464	0.092211	1.74017	0.085824
10 - 12	0.27253	0.126768	2.149828	0.034708
10 - 13	0.284455	0.128818	2.208186	0.030207
11 - 12	0.112066	0.087134	1.286131	0.202251
11 - 13	0.123991	0.111903	1.10803	0.271298
12 - 13	0.011925	0.074703	0.159634	0.873587

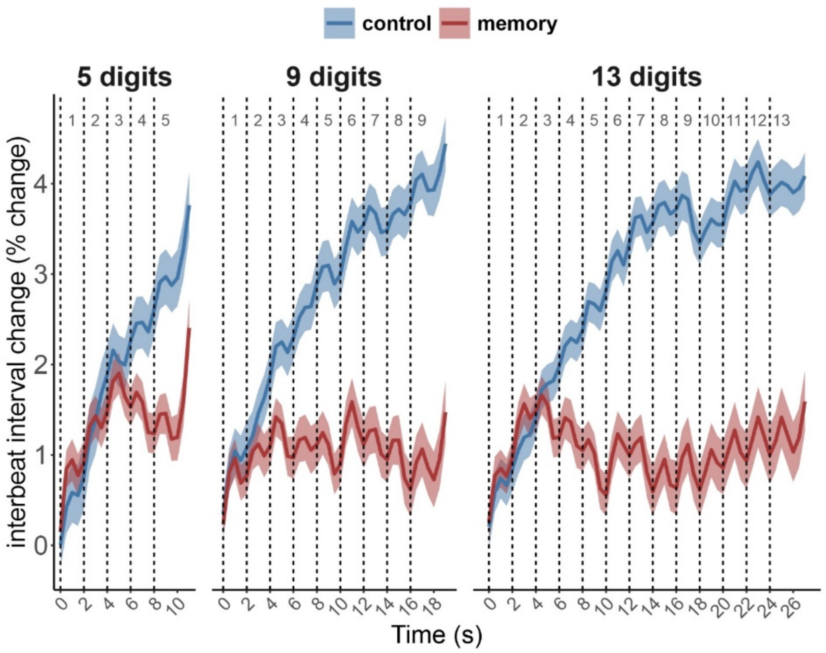

Figure S1 – Temporal dynamics of relative interbeat interval registered by ECG. Each dashed line represents the onset of a digit presentation. Time 0 indicates presentation of the first digit in the sequence.

Table S3 – The Task (control vs memory) and Load (13 levels) effects on relative interbeat intervals

	Interbeat interval			
	df	*F*	*p*	η^2
Task	1, 77	46.23	<.001	.38
Load	2.6, 199.98	19.74	<.001	. 20
Task x Load	3.4, 261.96	31.55	<.001	. 29

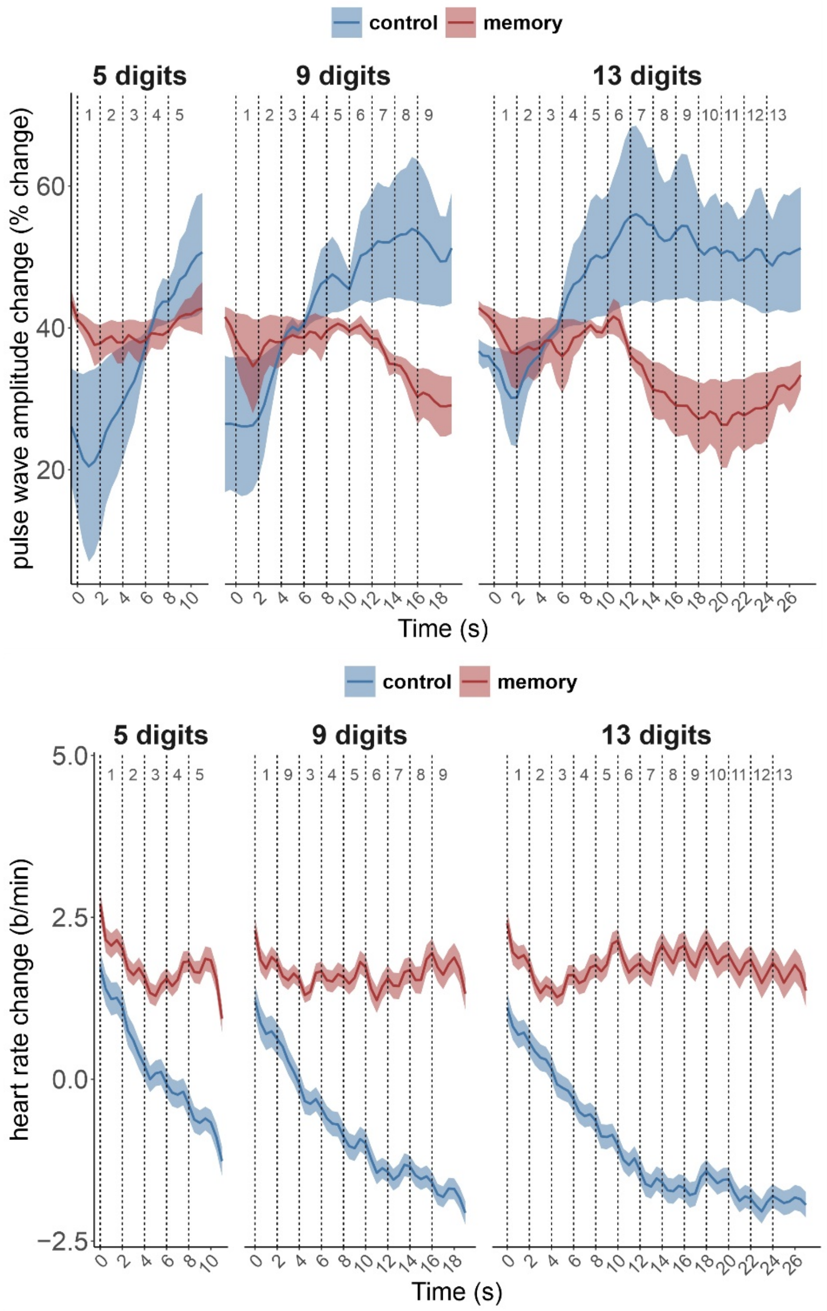

Figure S2 – Temporal dynamics of relative pulse wave amplitude (top panel) and heart rate (bottom panel) with resting state values used for baseline normalization. Each dashed line represents the onset of a digit presentation. Time 0 indicates presentation of the first digit in the sequence.

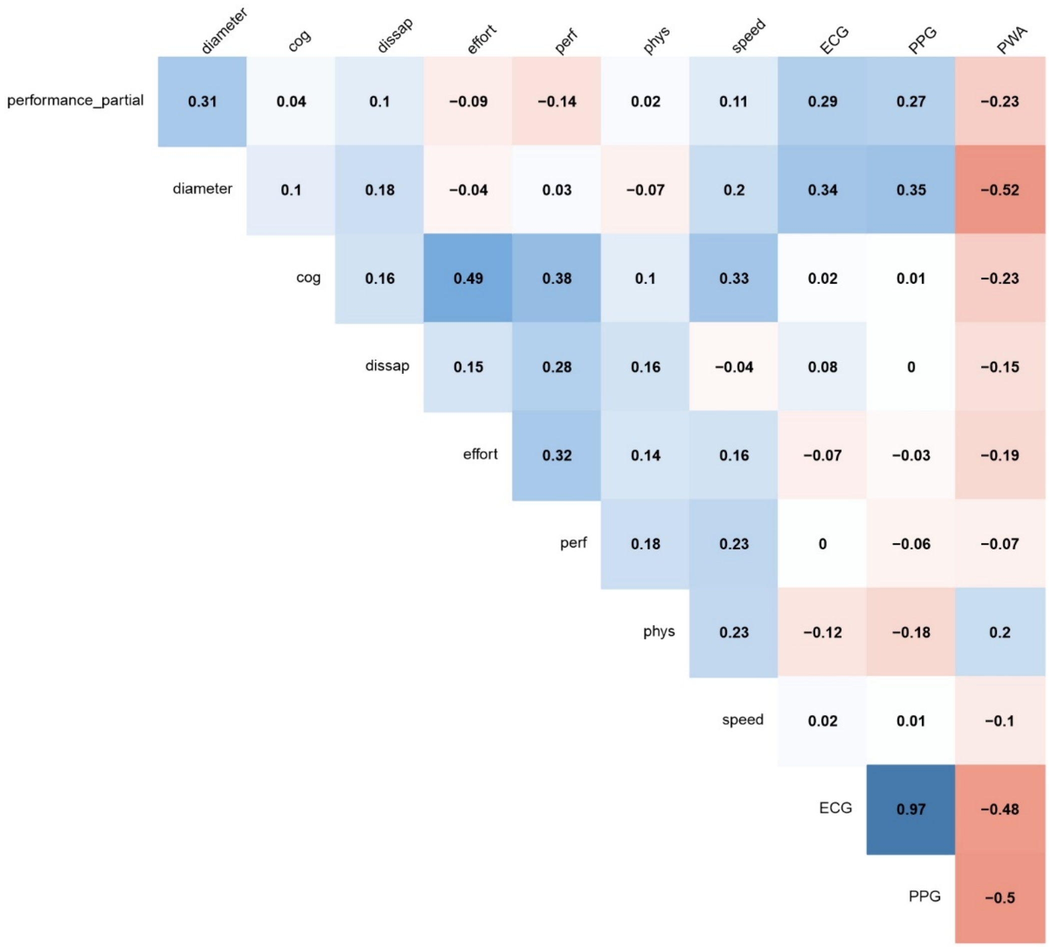

Figure S3 – Pearson correlations between physiological variables in the memory task: heart rate extracted from ECG – ECG, heart rate extracted from PPG – PPG, pulse wave amplitude – PWA, pupil size – diameter, behavioral performance (number of correctly recalled digits) – performance_partial, and NASA-TLX subscales (assessing how mentally demanding – cog, (2) frustrating – dissap, (3) effortful – effort, (4) difficult to perform – perf, (5) physically demanding – phys, and (6) temporally demanding – speed – the task was)
